# Deterministic generation and nanophotonic integration of 2D quantum emitters for advanced quantum photonic functionalities

**DOI:** 10.1515/nanoph-2024-0629

**Published:** 2025-01-24

**Authors:** Jae-Pil So

**Affiliations:** Department of Physics, 35016 Soongsil University, Seoul 06978, Republic of Korea; 35016 Integrative Institute of Basic Sciences, Soongsil University, Seoul 06978, Republic of Korea

**Keywords:** quantum emitter, 2D materials, nanophotonic integration, quantum photonics

## Abstract

Quantum emitters (QEs) are essential building blocks for quantum applications, such as quantum communication, quantum computing and metrology. Two-dimensional (2D) materials, such as transition metal dichalcogenides (TMDs) and hexagonal boron nitride (hBN), are promising platforms for scalable QE generation due to their unique properties, including their compatibility with external photonic structures. Advances in defect engineering and strain manipulation enable precise localization of emission sites within these materials, while integration with nanophotonic structures, including cavities and waveguides, enhances photon emission through the Purcell effect. This integration supports quantum functionalities like single-photon routing and spin-photon interactions. Challenges include achieving precise QE placement and emission control, as environmental factors can affect QE purity and indistinguishability. Nonetheless, electrically driven QEs, strain-tunable emission, and the integration of van der Waals magnets present opportunities for compact, scalable quantum devices with on-demand single-photon sources and spin-based quantum memory, positioning 2D QEs as foundational for next-generation quantum devices.

## 1 Introduction

Quantum technologies and quantum information science are rapidly emerging fields of research with a wide range of potential applications, as evidenced by foundational principles of quantum mechanics such as superposition and entanglement. These fields exploit the exotic and counterintuitive phenomena of quantum mechanics to perform tasks beyond the scope of classical physics. Among these phenomena, photon-mediated interactions at the single-photon level have opened up opportunities for advancements in quantum computation, simulation, and communication [1], [2], [3]. One of the most significant developments in this context is the emergence of deterministically generated quantum emitters (QEs) and their photonic integrations, which together form a critical foundation for future quantum technologies [4], [5]. The integration of QEs into larger quantum systems is a crucial step in the advancement of quantum technology [6]. Integrated quantum photonics, which involves the incorporation of nanophotonic elements such as cavities, waveguides and the others, plays a crucial role in their advanced quantum functionalities.

QEs, a system that can generate and emit photons one at a time on demand, are the backbone of many quantum technologies, including quantum key distribution, quantum computing, and quantum sensing [7], [8]. These emitters must adhere to stringent requirements, such as high purity, brightness, and indistinguishability, to function effectively within quantum systems. QEs can be broadly classified into two categories: excitonic emitters and defect-based emitters. Excitonic emitters, such as quantum dots, single molecules, and carbon nanotubes, generate photons through near-bandgap transitions [9]. While these systems have demonstrated high performance, they face challenges related to spatial and spectral inhomogeneity, which limit their scalability [10], [11]. Defect-based emitters, on the other hand, are often described as “artificial atoms” embedded in solid-state hosts. Common examples include color centers in wide-bandgap materials such as diamond and silicon carbide. These defect-based systems offer the advantage of more precise control over the position and spin characteristics of the emitter which can provide quantum degrees of freedom [12], [13], [14]. However, they present their own set of challenges, particularly in terms of integration with photonic devices and photon extraction efficiency.

To this end, QEs in two-dimensional (2D) materials have emerged as a promising platform for advancing quantum technologies, offering unique properties that are unattainable in bulk materials [10], [11], [15]. The discovery of QEs in 2D materials, such as transition metal dichalcogenides (TMDs) and hexagonal boron nitride (hBN), has opened new avenues for research in quantum photonics [16]. These materials exhibit quantum properties that are typically absent in their bulk counterparts, including large exciton binding energies, tunable electronic properties, and the ability to host localized QEs.

The first reports of QEs in 2D materials came in 2015, with concurrent discoveries in monolayer tungsten diselenide (WSe_2_) and other TMDs [17], [18], [19], [20]. QEs in TMDs typically exhibit sharp, localized emission lines in their photoluminescence (PL) spectra, often red-shifted from the free exciton emission [21], [22], [23], [24]. These emissions are attributed to excitons trapped in localized states, possibly due to crystallographic defects or strain-induced confinement in the 2D material [15], [16]. These QEs are highly promising due to their narrow linewidths (typically 0.1–0.7 meV) and their ability to generate single photons on demand, which is a critical requirement for quantum technologies. More interestingly, moiré trapped excitons arise in 2D heterostructures where two different layers of TMDs are stacked with a slight twist or lattice mismatch, creating a periodic moiré pattern. These moiré superlattices generate localized potential wells that can trap excitons, leading to the formation of QEs [25], [26]. These excitons exhibit unique properties, such as long lifetimes, tunable emission energy, and strong interaction with external fields like electric or magnetic fields.

Similarly, hBN, an insulating 2D material, has also been found to host QEs with properties comparable to TMD-based emitters [27], [28], [29], [30], [31]. QEs in hBN are typically associated with deep-level defects within the bandgap, and they have the distinct advantage of operating at room temperature, making them highly attractive for practical quantum photonic devices. While the exact nature of the defects responsible for quantum emission in hBN is still under investigation, with candidates ranging from carbon-related defects to boron vacancies, hBN QEs have recently garnered significant attention due to their unique optical and spin properties, making them attractive candidates for quantum technologies, particularly in quantum sensing and information processing [32], [33], [34], [35], [36].

A remarkable aspect of hBN QEs is the presence of optically addressable spin states, which were first demonstrated through optically detected magnetic resonance (ODMR) at room temperature by Gottscholl et al. [37]. The most notable spin defect in hBN is the negatively charged boron vacancy (*V*_B_), a spin-1 system. This defect ensemble exhibits a ground state triplet, which is split by a zero-field splitting of approximately 3.47 GHz and further hyperfine interactions with surrounding nitrogen nuclei. These spin properties make the *V*_B_ center in hBN analogous to the nitrogen-vacancy (NV) center in diamond, offering the potential for spin-based quantum applications at room temperature [13], [38]. Notably, the *V*_B_ center in hBN can be created through ion implantation or electron beam irradiation, making it possible to generate these defects deterministically. Further research has shown that the spin coherence time (T2) of these defects is in the microsecond range, making them suitable for high-sensitivity quantum sensing [39]. More recently, ODMR of the single defects in hBN which have recently been assigned to carbon impurities were demonstrated [40]. The emission from these single defects exhibits ultra-high brightness. While the spin-related research on hBN is still in its early stages, these findings highlight the potential of hBN for scalable quantum technologies, particularly in spin-photon interfaces and room-temperature quantum sensing.

On the other hand, integration of QEs with nanophotonic structures is crucial for quantum photonic multi-functionalities. QEs such as color centers in diamond, 4H-SiC or semiconductor quantum dots benefit greatly from coupling with photonic structures, including cavities and waveguides [41], [42], [43], [44]. These nanophotonic structures facilitate enhanced light–matter interactions, which are essential for efficient photon emission, through the Purcell effect. For example, color centers in 4H-SiC, when coupled to high quality factor (Q) photonic cavities, show significantly enhanced emission due to increased photon collection efficiency and improved quantum coherence [42], [45]. Moreover, waveguide-coupled emitters provide high efficiency for on-chip quantum light processing, enabling photons to be guided and processed within integrated circuits [44], [46]. This platform, as demonstrated in recent works, allows for the off-chip coupling of QEs with minimal losses, thereby making it feasible for practical quantum network applications.

**Figure 1:**
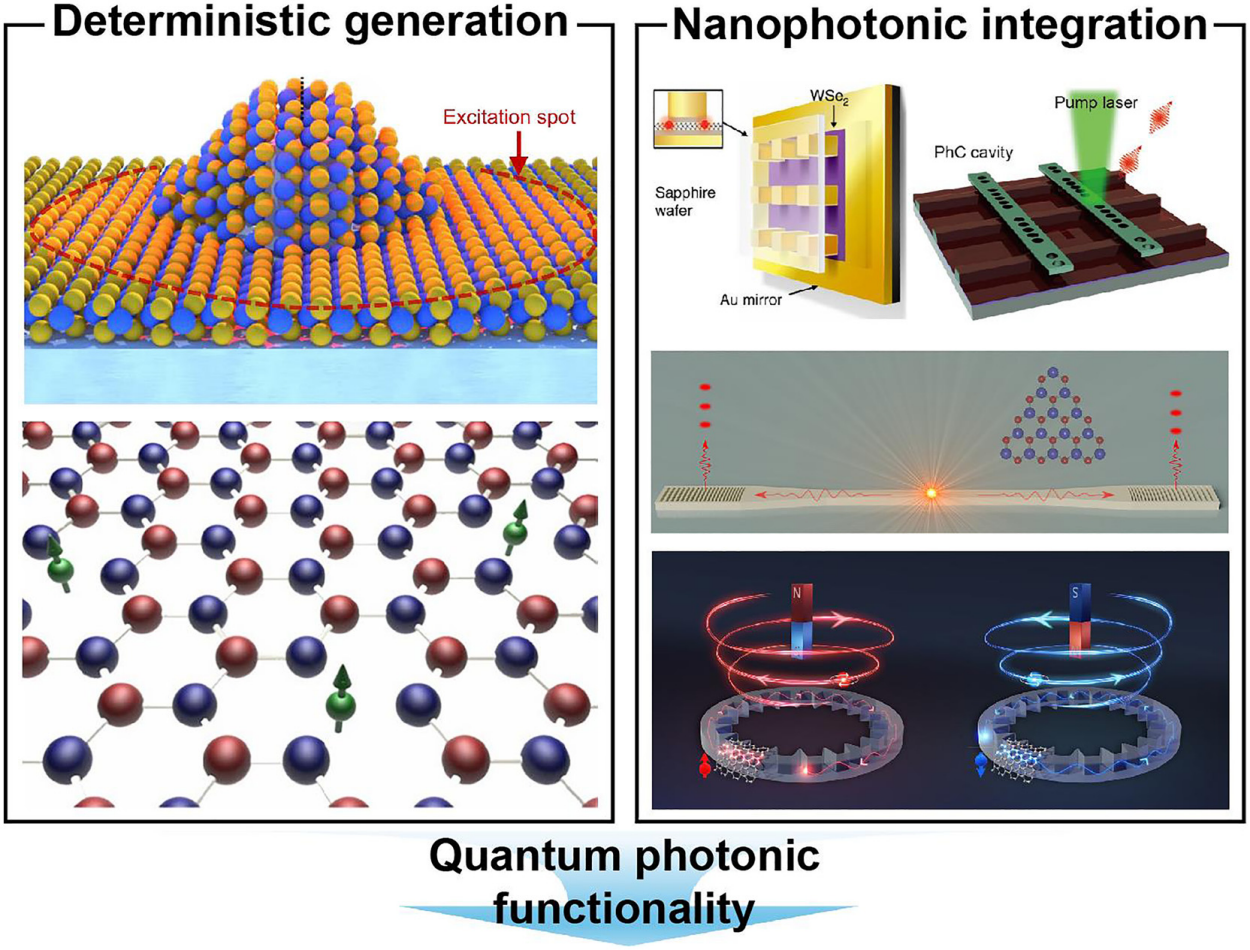
Schematic of deterministic generation and nanophotonic integration of 2D QEs for advanced quantum photonic functionalities. Reproduced with permission from Refs. [37], [53], [54], Nature Publishing Group, Refs. [55], [56], American Chemical Society. Ref. [50], American Physical Society.

This field of research has rapidly gained momentum due to the distinctive potential of 2D materials to host QEs with adjustable physical properties and their capacity to be integrated into nanoscale structures. Effective integration can lead to not only enhanced emissions, but also new functionalities such as photonic quantum gating [47], [48], spin-photon interaction [49], single-photon routing [50], and the development of scalable quantum devices [51], [52]. Nevertheless, two significant challenges remain: the first is the necessity to achieve deterministic generation of emitters, which ensures precise control over their placement and behaviour. The second is the optimization of their coupling with nanophotonic structures. It is imperative to overcome these challenges to advance quantum technologies (Figure 1). In this perspective, we discuss three main categories regarding the generation and the integration of QEs in 2D materials with nanophotonics: (1) the deterministic generation of 2D QEs, (2) the functionalities of nanophotonic integrated 2D QEs, and (3) the challenges and the prospects of the integration of 2D QEs with nanophotonic structures.

## 2 Deterministic generation of 2D QEs

The deterministic creation of QEs in solid-state materials is a crucial step in the development of scalable photonic applications. These emitters offer a significant advantage over atomic or ion-based counterparts, as they can be integrated with external structures with greater ease. To achieve this, it is necessary to develop a method for the precise generation of QEs at desired locations with a high yield is necessary. In semiconductor quantum dots, lithographic techniques facilitate the growth of nanostructures at specific sites, thereby enabling the creation of on-demand single-photon sources with near-unity brightness [57], [58]. In case of wide-bandgap materials, atomic defects such as diamond NV centers can be produced using implantation or irradiation [59], [60], [61]. Similar techniques have been developed for 2D materials, employing defect implantation or strain-induced exciton confinement to control emitter positioning [16], [62]. These developments in deterministic QE creation hold promise for advancing solid-state quantum technologies.

### 2.1 Defect implantation

Recent progress on deterministic creation of excitonic QE in TMDs, including WS_2_ and MoS_2_, has been demonstrated using helium-ion beam irradiation to generate optically active defect centers. These defect emitters exhibit distinctive PL characteristics, namely highly localized and spectrally narrow, which makes them suitable for applications in single-photon sources and other quantum technologies with precise spatial and spectral control. Most of studies focused on monolayer semiconductors, where helium-ion beam irradiation in small, controlled regions created defect emitters that emit light at an energy of 100–220 meV below the free exciton energy [63], [64], [65], [66]. The use of hBN encapsulation minimized linewidth broadening, yielding stable, localized emission centers with narrow spectral features. This approach demonstrated the on-demand positioning of photon emitters in MoS_2_ monolayers, achieving an emitter yield of up to 18 % per irradiated site and exhibiting single-photon emission verified by antibunching behavior (*g* ^2^(0) ∼ 0.23–0.27) [64].

The study of QE creation via defect implantation in TMDs has provided insights into the fundamental role of atomic defects in these materials. By using far-field optical spectroscopy, atomic-resolution scanning probe microscopy, and ab initio theory, optical defect emissions which arise from pristine sulfur vacancies in MoS_2_ monolayers have been demonstrated [67]. By encapsulating MoS_2_ with hBN and applying mild annealing, the typical broad L-band luminescence from adsorbates was suppressed, underscoring the significance of maintaining the vacancy’s pristine nature. In addition, further investigations into these emitters, utilizing high-field magneto-PL spectroscopy, revealed detailed electronic and magnetic characteristics of He-ion induced sulfur vacancies [68]. The defect emission lines exhibited valley-Zeeman splitting, indicating spin-valley selectivity and zero-field spin splitting, which are advantageous for quantum information applications. These findings are supported by ab initio calculations, which suggest the emissions result from transitions between in-gap defect states and the MoS_2_ valence band, while the counterpart emissions are postulated to correspond to a chemically functionalized defect. Together, these findings highlight the role of controlled defect engineering in TMDs, particularly MoS_2_, where precise manipulation of sulfur vacancies can lead to stable, tunable single-photon sources with potential applications in spin-based quantum technologies.

On the other hand, recent studies have advanced the use of defect implantation in hBN to create high-purity QEs, with promising parallels to diamond NV centers. Techniques such as helium-ion beam [69], [70], [71], electron-beam [29], [72], [73], and laser writing [74] enable the mask-free and site-specific generation of atomic defects in hBN. These methods have been demonstrated to produce tunable single-photon emissions at room temperature with high stability and spectral purity, rendering hBN emitters suitable for scalable photonic integration without the necessity of additional structural supports. In particular, QEs in hBN fabricated through focused helium-ion beams have been demonstrated to exhibit high levels of positional accuracy (below 10 nm) and reproducibility. These emitters display high brightness and photostability, which are essential for practical quantum applications. By avoiding oxygen passivation and other external adsorbates, the pristine defect states in hBN emitters ensure high spectral purity, a pivotal prerequisite for reliable quantum communication protocols.

Moreover, research on hBN QEs has increasingly focused on elucidating the origins of these emitters and how distinct defect species influence their properties. This has entailed the integration of controlled defect implantation with advanced crystal growth and spectroscopy techniques [75], [76], [77], [78], [79]. Along with ion implantation, metal–organic vapor-phase epitaxy (MOVPE) and molecular beam epitaxy (MBE), researchers have investigated the role of carbon as a critical factor in the generation of QEs in hBN [76]. The findings of this study indicate that carbon implantation in hBN is a crucial for achieving visible single-photon emission. Higher carbon concentrations result in the formation of ensembles of emitters and lower concentrations, yielding isolated defects that demonstrate ODMR at room temperature [40]. Complementary work used resonant inelastic X-ray scattering and PL spectroscopy to explore electronic excitation states within defective hBN [78]. This study revealed a fundamental excitation at 285 meV associated with harmonics tied to SPEs, highlighting the role of nitrogen anti-bonding (N π*) orbitals in shaping the electronic states of QEs. These elementary excitations contribute to a deeper understanding of quantum emission in low-dimensional materials and point to the potential of hBN as a tunable platform for quantum photonics.

On the other hand, the other study suggested that QEs in hBN emitting around 2 eV may originate from organic molecules, particularly polycyclic aromatic hydrocarbons (PAHs) [80]. Experimental evidence indicates that emission centers form during high-temperature processing in the presence of organic residues, and their photoluminescence spectra resemble PAH fluorescence. These emitters are typically located at the hBN/substrate interface, where trapped organic molecules may interact with hBN defect sites. This model explains variations in emission properties and aligns with behaviors such as Stark shifts and vibrational features, previously attributed to hBN defects. These findings imply a significant role for organic contaminants in SPE formation. Research into these diverse origins of SPEs continues to advance, exploring both intrinsic defects and extrinsic factors.

### 2.2 Strain engineering

The high Young’s modulus and elastic strain limits of 2D materials make them ideal for precise strain engineering [62], [81]. Research on QE creation in TMDs has evolved significantly since the discovery of randomly distributed QEs [17], [18], [19], [20]. Early observations suggested a correlation between localized strain in these materials and the emergence of SPEs [82], [83]. This hypothesis was confirmed experimentally by Kumar et al., who demonstrated that strain regions in TMDs had a higher likelihood of producing SPEs, with spectral properties sensitive to strain magnitude. Subsequent studies achieved greater precision and reproducibility in QE creation using strain engineering techniques. Branny and Palacios–Berraquero’s works in 2016 marked a notable advancement when they applied strain to TMDs by stamping them onto nano-pillars, which acted as nanostressors to create highly localized strain [84], [85]. This method yields near-unity QE creation, with up to 95 % purity and detection rates reaching 10 kHz. Although the QEs were generated effectively via this approach, they displayed a broad spectral range from 720 to 800 nm and required cryogenic temperatures for stability. Subsequent studies have explored alternative strain-engineering approaches using metallic nanocubes [53], nanoparticles [86], [87], nanogaps [88], [55], nano-indentation with AFM tips [89] and the other novel approaches [90], [91], [92].

**Figure 2:**
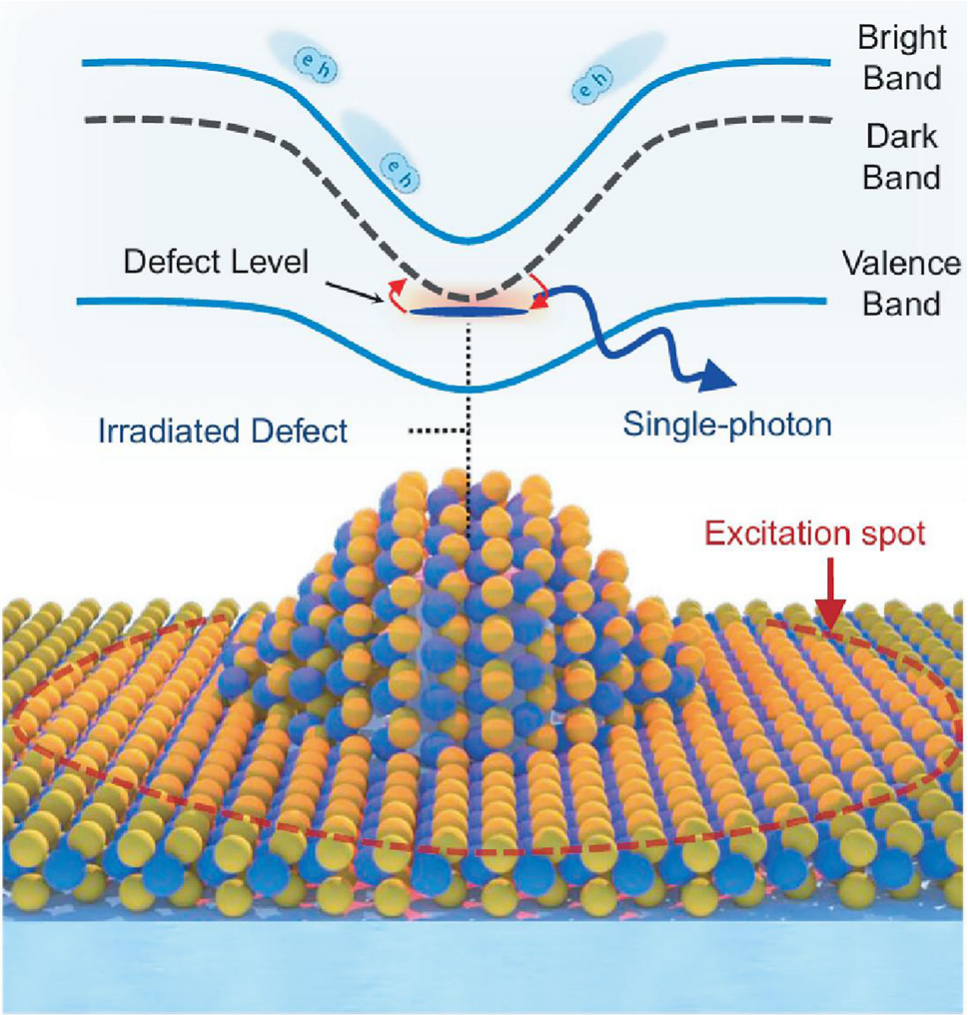
Schematic of a strain-induced single-photon emitter and its corresponding band structures. Spatial bandgap variations caused by strain create potential wells, directing excitons toward these low-energy regions. When a monolayer TMD is strained over a nanostructure, with defects added at the top, dark excitons are modified to form intervalley defect excitons. This configuration disrupts the spin-momentum locking, allowing dark excitons to recombine via the defect, resulting in bright single-photon emission. Reproduced with permission from Ref. [54], Nature Publishing Group.

Recognizing that strain alone did not fully account for the quantum emissions, researchers initiated an investigation into the role of atomic defects in the creation and properties of SPEs in TMDs (Figure 2). Theoretical studies have proposed that both defects and strain are crucial for understanding the quantum emission, particularly in explaining their brightness [83]. Experimental results have supported this dual-factor model [54]. By employing a combination of electron beam (e-beam) irradiation to introduce defects and nano-pillars to apply strain in WSe_2_ monolayers, researchers were able to independently control the contributions of defects and strain in SPE creation. The results demonstrated that e-beam induced defects exhibited emission energies that were at 100–150 meV lower than those observed from strain-induced emitters. Furthermore, it was also found that reducing the density of defects in WSe_2_ monolayers improved the operational temperature of devices, creating higher thermal barriers and enabling functionality up to 150 K. Emitters that are generated through this method exhibited 95 % purity, with some showing exciton-biexciton cascades, suggesting the potential for high-quality QEs with enhanced functionality.

**Figure 3:**
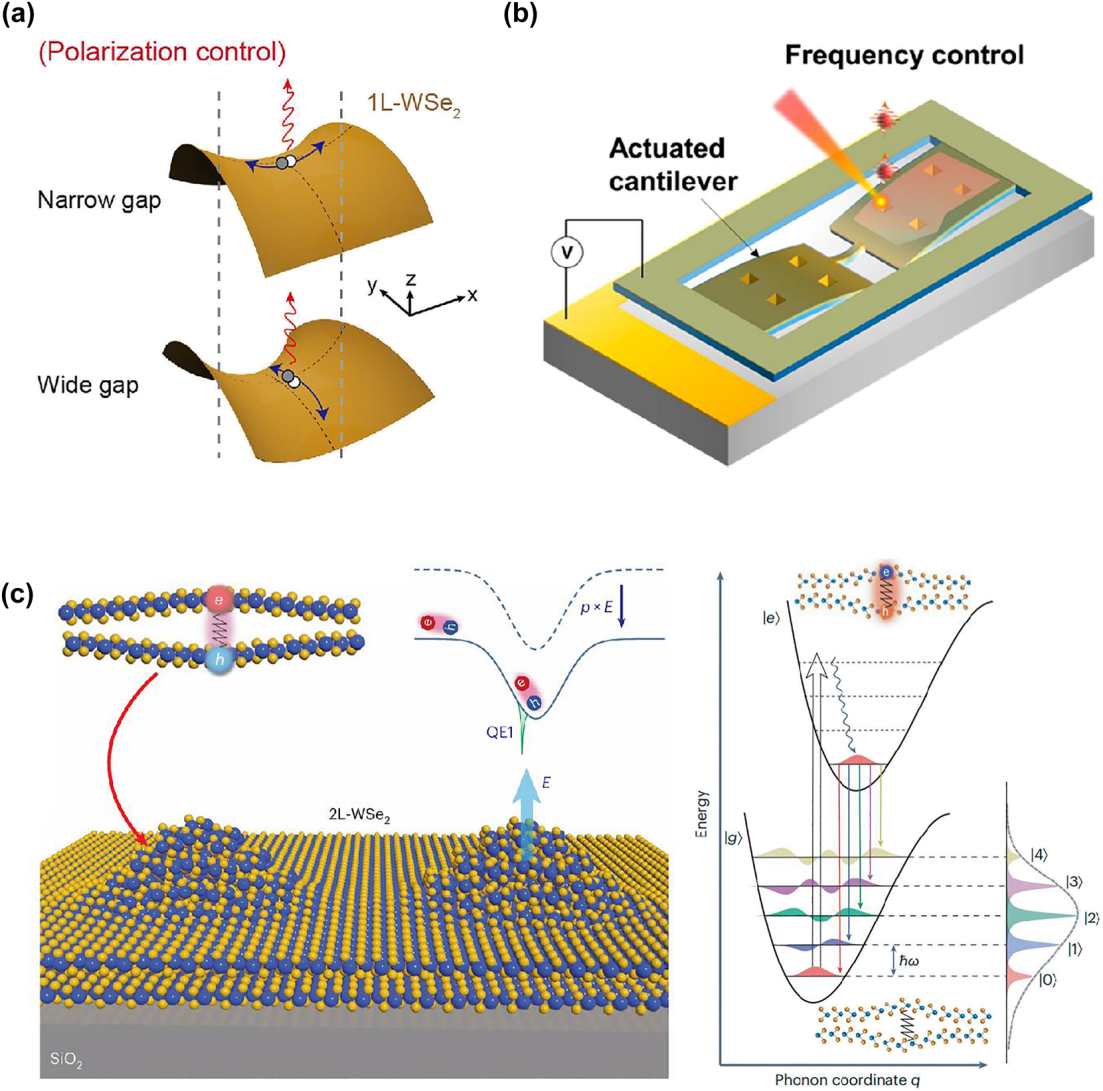
Tuning the emission properties using strain-engineering. (a) Schematic of the polarization control of QEs using a nanogap structure. The size of the nanogap affects the strain directions of a monolayer WSe_2_, and the exciton oscillation is formed elongated along this direction. (b) Schematic of strain-controlling platform based on actuated microcantilever. Actively controlled strain via voltage actuation tunes the emission frequency. (c) Schematic of the electrically tunable phonon–photon coupling in strain-engineered QEs in bilayer WSe_2_. The interlayer exciton with vertical dipole moment and breathing-mode of phonons interact each other, resulting in direct modulation of the emission energy. The energy level diagram shows the phonon replica lines corresponding to the coupling between a single interlayer exciton and single-phonon modes that are colocalized in the QE. Reproduced with permission from Refs. [55], [95], American Chemical Society (a and b), and Ref. [97], Nature Publishing Group (c).

These methods offered dynamic strain control and deterministic generation of QEs; however, consistent limitations across these approaches were the lack of control over the emission properties, such as an emission energy, and a polarization orientation, which are essential to achieve strongly coupled QEs with nanophotonic structures. To this end, advanced strain engineering techniques offer a versatile approach to creating tunable QEs with controlled emission properties [93]. Strain engineering can be employed to control the polarization of emitted photons [55], [94]. A local strain gradient manipulates the band structure to create a trapping potential at the strained regions. For example, by adjusting the nanogap size, researchers can control the directional elongation of the potential well, thereby allowing polarization-specific photon emission (Figure 3a). This approach provides deterministic control over both position and polarization of the emitter, which is essential for the development of reliable, tunable single-photon sources based on TMDC materials. A further noteworthy aspect of strain engineering is its ability to control the emission frequency of QEs in WSe_2_ [95], [96]. By integrating WSe_2_ on a nanopatterned microcantilever, researchers can dynamically adjust local strain, tuning the QE emission frequency by up to 3.5 meV within a strain range of 0.07 % (Figure 3b). Furthermore, the integrated QE also exhibited a reduction in fine-structure splitting by 11 %, providing finer control over the QE’s spectral characteristics. This dynamic tuning compensates for the inherent frequency variations in WSe_2_ QEs, making them more consistent and predictable for applications. The microcantilever platform offers a promising foundation for frequency control, with the potential for expanding the tuning range by increasing the strain limits within the device. Strain engineering has also been employed to control the emission frequency of hBN QEs [69]. By transferring hBN films onto a flexible polycarbonate beam, researchers were able to apply controlled tensile strain, achieving frequency shifts in the range of −3 to 6 meV/% by bending the substrate.

Beyond frequency and polarization control, strain engineering also allows for the manipulation of phononic coupling in QEs [97]. This was demonstrated in bilayer WSe_2_, by achieving coupling between phonons and photons. In these bilayer QEs, a strong phonon–photon coupling was observed, manifesting as multiple phonon replica lines in the single-photon emission spectra (Figure 3c). This phonon coupling is a consequence of the colocalization of interlayer excitons and breathing-mode phonons, leading to a tunable Huang–Rhys factor – the highest reported in solid-state QE systems to date. This high phonon–photon coupling provides a mechanism to modulate exciton energy directly through phononic interactions, adding an internal mechanical degree of freedom for quantum information processing. Such systems present exciting possibilities for engineering quantum light sources with tunable phononic interactions, advancing the field of quantum photonics and optomechanics.

## 3 Integration of QEs with nanophotonic structures

The implementation of QE-based quantum photonic applications hinges on the integration of QEs with nanophotonic structures, which is essential for advancing the control of light–matter interactions. This integration enables the precise manipulation of QEs by altering their electromagnetic environment, thereby enhancing the emission efficiency or spatial control, and incorporating optical circuits [6], [16], [62]. Nanophotonic resonators confine light at scales smaller than the wavelength, significantly modifying the emission dynamics [98]. Additionally, advances in nanophotonics have enabled full control over emission direction, phase and polarization, which are particularly valuable in the realization of coherent, indistinguishable photon sources for secure communication. The integration of QEs based on 2D materials with nanophotonic systems offers a promising avenue for the development of efficient and versatile quantum light sources and quantum photonic devices.

### 3.1 Plasmonic cavity

Plasmonic cavities have emerged as a promising method to achieve Purcell enhancement in 2D QEs. The Purcell factor is optimally determined by the cavity’s quality factor (Q) and inversely by the mode volume (*V*_mode_). Although plasmonic cavities inherently a have low Q due to high losses, their ability to generate ultra-small mode volumes compensates for this, allowing for a significant Purcell enhancement. Localized plasmonic field enhancements at the edges and sharp geometries of nanostructures offer high field confinement, which is well-suited for matching with strain-localized 2D material QEs, such as those in TMD and hBN. For instance, hBN-based QEs placed on silver nanoparticle arrays demonstrated an increased saturation intensity by 2.6 times, with an overall Purcell factor of approximately 2 [99], [100]. In the case of TMD-based QEs, enhanced emission has been demonstrated by coupling them to plasmonic nanostructures, such as metal nanorods [101], and nanocones [102], which can also be used as strain-inducing structures. Such systems benefit from both localized strain induction, which activates quantum emission, and the ability to couple directly with the plasmonic field without needing additional alignment steps. Moreover, due to the randomly occurring emission wavelengths of TMD QEs, a moderate Q factor in the plasmonic cavity is often preferable, as it provides a wider resonance range, accommodating the varied emission wavelengths of QEs.

**Figure 4:**
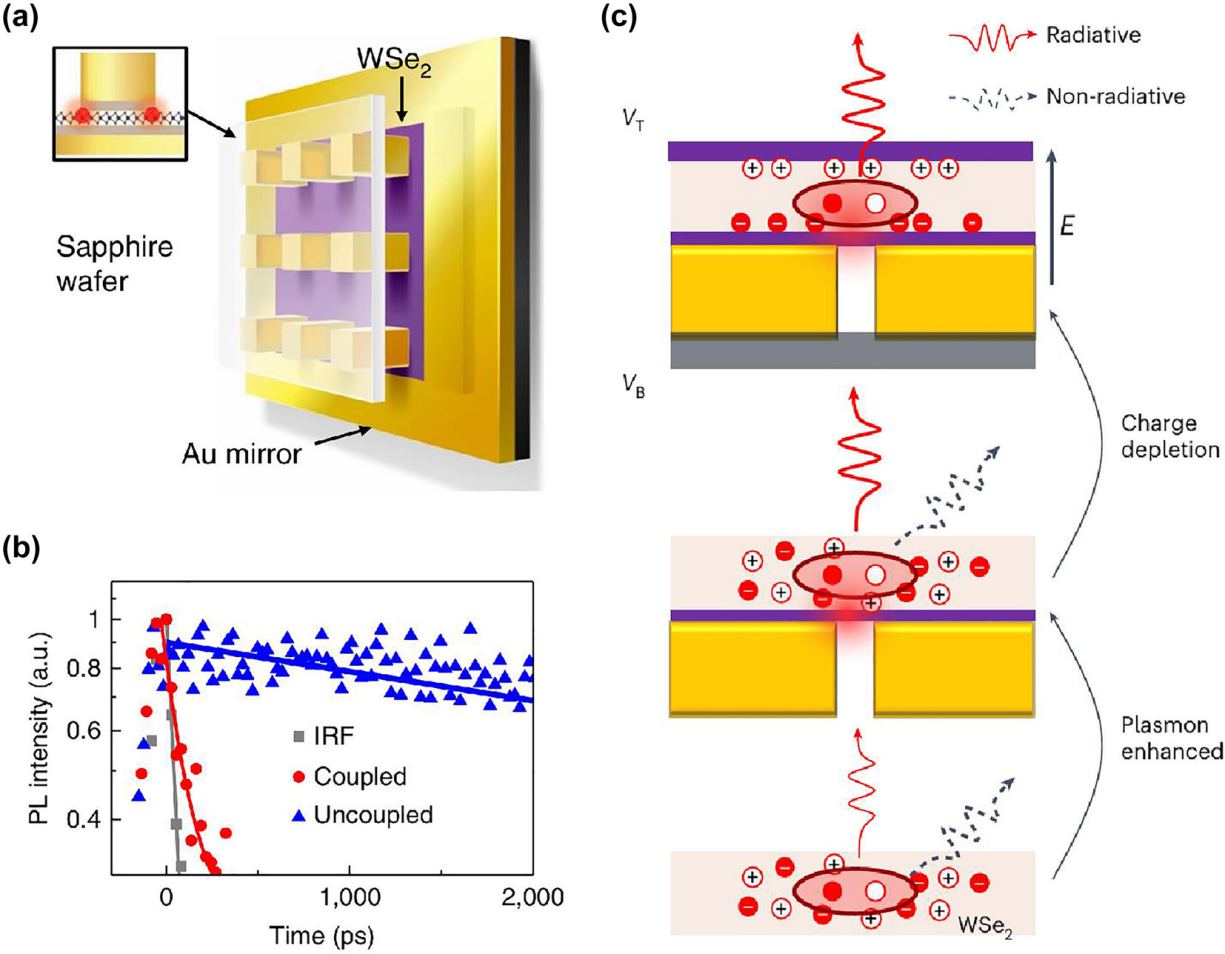
QEs coupled to plasmonic cavities. (a) Schematic of a monolayer WSe2 coupled to a plasmonic gold nanocube array and a planar mirror-like substrate. Al_2_O_3_ spacer on each side prevents optical quenching and short circuiting of the plasmonic gap mode. (b) Spontaneous emission lifetime measurements yielding a significant decay rate enhancement of 57. (c) Schematic of design and working principle of charge-depletion-enhanced QEs coupled with plasmonic nanogap cavity. Plasmonic resonance increases the local density of state via the Purcell effect, and charge depletion by applying an electric field in active medium results in reduced non-radiative decay. Reproduced with permission from Refs. [53], [104], Nature Publishing Group.

Milestone research in the field has been reported by Luo et al. [53]. The deterministic creation and coupling between QEs and plasmonic modes were achieved by transferring a monolayer WSe_2_ onto a metallic mirror and then placing a metal nanocube on top of it (Figure 4a). Both electric field enhancement and strain-induced exciton localization occur at the sharp corners of a metal nanocube, resulting in the Purcell factor of up to 551, an average of 181 (Figure 4b), single-photon emission rates reaching 42 MHz, and exciton linewidths as narrow as 55 μeV. In addition, by using flux-grown WSe_2_, the exciton lifetime was extended to 14 ns, and cavity-enhanced quantum yields increased significantly from 1 % to an average of 44 % (up to 65 %). The lithographically defined emitter positioning and high quantum yield make this plasmonic nanogap array approach an ideal platform for efficient integration platform [103]. Furthermore, in order to achieve near-unity quantum efficiency, it is necessary to significantly suppress non-radiative decay pathways, which presents a considerable challenge in the field. More recently, QEs with a near-unity quantum efficiency have been demonstrated by using both plasmonic enhancement and dual-gate charge depletion [104]. In this approach, QEs were generated within gold nanogaps, thereby enhancing the radiative decay rate by increasing the local density of states. This enhancement resulted in a brightness increase of over 20 times compared to that of uncoupled emissions. The breakthrough of this research lies in the second step: suppressing non-radiative decay through an applied electric field using a dual-gate configuration. By applying a large electric field, charges were depleted from the region surrounding the excitons, effectively reducing exciton-charge interactions that typically lead to non-radiative decay (Figure 4c). This dual-gate configuration allowed precise control over charge redistribution without relying solely on electrical doping. As a result, the quantum efficiency of the emissions reached up to 90 %, with some emitters approaching unity efficiency. Additionally, PL linewidths narrowed during the intensity enhancement, indicating a reduction in spectral broadening and approaching lifetime-limited linewidths, which are crucial for quantum applications that require high coherence.

### 3.2 Dielectric cavity

The integration of QEs with dielectric optical cavities has also garnered significant attention due to its potential to enhance light–matter interactions, which are of critical importance for the advancement of quantum photonic technologies. Dielectric photonic crystal cavities (PCCs) are particularly advantageous due to their high-Q factors and low losses, facilitating efficient Purcell enhancement. In contrast to plasmonic cavities, which suffer from high losses despite their ultra-small mode volumes, dielectric PCCs enable substantial Purcell enhancement through their high Q factors. Nevertheless, integrating TMD QEs with PCCs has proven to be a significant challenge. Notably, several works demonstrated coupling of a TMD QEs to dielectric cavities, such as nanobeam PCC [55] and nano-antennas [105], [106].

Meanwhile, it is noteworthy that hBN QEs are particularly compatible with dielectric structures such as micro-cavity [107], [108], and bullseye cavity [109], [110]. These approaches can be employed to enhance the light extraction efficiency of QEs embedded in host materials with a thickness of typically tens to hundreds of nanometres. In addition, the emissions from deep-level defects within hBN exhibit consistent zero-phonon line (ZPL) wavelengths, simplifying integration with dielectric high-Q cavities. This consistency serves to mitigate the challenges associated with the narrow spectral resonances of high-Q cavities, which typically require precise spectral alignment with the emitter. Recent advancements have demonstrated the successful coupling of hBN quantum emitters with various dielectric PCCs [111], [112], [113], [114]. For example, researchers have integrated hBN emitters into PCCs, resulting in enhanced emission rates and improved photon extraction efficiency. Such integration leverages the high-Q factors of dielectric cavities to boost the radiative decay rates of the emitters, leading to brighter and more coherent single-photon sources.

In terms of device fabrication methodologies, two primary approaches exist for the integration of QEs with dielectric cavities: hybrid and monolithic. In the hybrid method, the emitter is placed onto a cavity made from a different material. In contrast, the monolithic approach involves embedding the emitter within a nanostructure fabricated from the same material. The monolithic method is particularly effective for hBN emitters. The monolithic fabrication approach can be employed to create compact and efficient quantum photonic devices [114]. The monolithic fabrication approach, whereby both the emitter and the cavity are fabricated from the same material, has the advantage of reducing losses at material interfaces and enhancing device performance. In the case of hBN, monolithic fabrication involves the etching of nanostructures directly into hBN flakes, resulting in integrated systems with reduced imperfections and improved quality factors. These developments underscore the potential of combining 2D QEs with optical cavities to create efficient, scalable, and high-performance quantum photonic devices.

### 3.3 Waveguide

For scalable quantum photonic applications, the integration of QEs with waveguides is a crucial step in directing quantum light precisely within photonic circuits. These quantum photonic circuits serve as the foundational elements for multi-functional quantum applications. The integration of 2D QEs with waveguides is a particularly promising avenue of research due to the unique characteristics of 2D materials, such as their tunability and compatibility with photonic structures. A variety of approaches have been explored to couple 2D QEs with waveguides. Among these, plasmonic waveguides offer both Purcell enhancement and photon guiding, which is a result of the strong field confinement in plasmonic waveguide modes at metal–dielectric interfaces, such as metal nanowires [115], [116], and slot waveguides [117], [118]. However, plasmonic waveguides are subject to intrinsic non-radiative losses in metals, which limit their scalability for large-scale quantum circuits.

**Figure 5:**
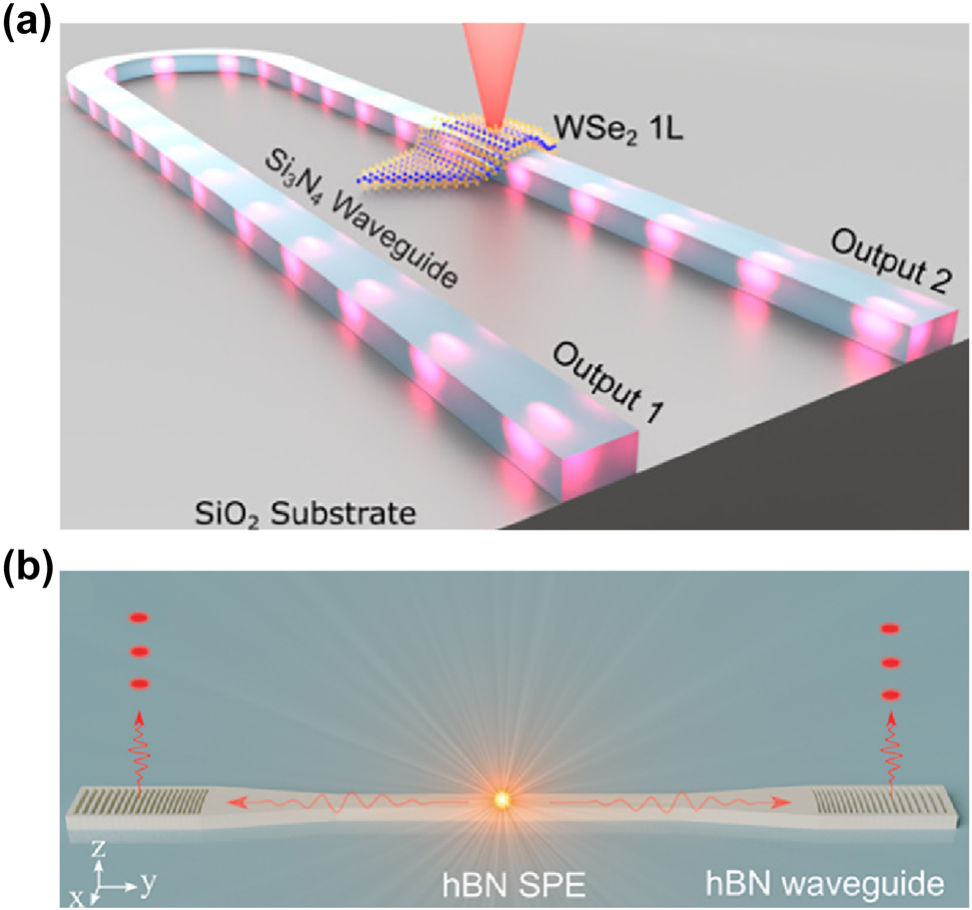
Waveguide-coupled 2D QEs. (a) Schematic of a coupled monolayer WSe_2_ QE with a U-shaped Si_3_N_4_ waveguide on a SiO_2_ bottom cladding. (b) Schematic of a hBN QE in a monolithic hBN waveguide. Reproduced with permission from Refs. [121], 56], American Chemical Society.

To address this limitation, researchers have shifted focus toward dielectric waveguides, which offer high transmission efficiency without the non-radiative decay pathways associated with plasmonic structures. Dielectric waveguides, particularly those based on materials such as silicon nitride (SiN) and aluminum nitride (AlN), have proven to be effective for integrating 2D materials. Recent studies have demonstrated successful integration of WSe_2_ and hBN with dielectric waveguides, achieving high-efficiency single-photon emission [56], [119], [120], [121], [122], [123]. For example, monolayer WSe_2_ has been coupled with SiN waveguides, where the strain-induced localization of emitters at waveguide edges allowed for effective coupling and controlled single-photon emission [121]. In this noteworthy study, the researchers fabricated a U-shaped Si_3_N_4_ waveguide with a WSe_2_ monolayer positioned at the waveguide’s edge (Figure 5a). This configuration enabled multiplexed single-photon emission along the waveguide, with the QEs exhibiting single-photon purity values of approximately 0.15 and resonance fluorescence with a *g* ^(2)^(0) of 0.377 in the absence of background substraction. The arrangement allowed for simultaneous waveguide coupling and controlled excitation, indicating a significant step toward scalable, on-chip quantum photonic integrated circuits (PICs). The positioning of the QEs at the edge of waveguide resulted in optimized coupling efficiency and directed the photon emission into the waveguide mode with greater efficiency [122].

Furthermore, QEs in hBN can be integrated with waveguides via hybrid or monolithic approaches, each of which offers distinct advantages. In a hybrid integration approach, the hBN layer containing the QEs is positioned atop a waveguide fabricated from a different material [123]. This approach allows flexibility in combining materials but may lead to inefficiencies at the material interface. In contrast, the monolithic approach involves fabricating the waveguide and quantum emitter directly within the same hBN material, resulting in higher coupling efficiency and simplified fabrication steps [56] (Figure 5b). For instance, finite-difference time-domain (FDTD) simulations have demonstrated that QEs embedded within a monolithic waveguide can achieve a coupling efficiency of 0.4, surpassing the efficiencies of 0.04 and 0.11 observed in hybrid and surface-mounted configurations, respectively. This improvement results from the precise spatial overlap of the optical mode with the emitter, which is challenging to achieve with hybrid methods due to potential misalignment and material losses at the interface. Monolithic waveguides have been fabricated with structures such as slot waveguides and integrated grating couplers, facilitating efficient photon emission and routing. These waveguides are designed to enhance the interaction between the waveguide mode and the embedded emitters, allowing light to be effectively directed toward on-chip photonic components such as grating couplers.

Looking ahead, improvements in the integration of 2D QEs with waveguides will facilitate more complex quantum photonic circuits by providing scalable, highly efficient, and versatile platforms for single-photon routing. These advances will provide scalable, highly efficient, and versatile platforms for routing single photons. Improvements in photon coherence and directionality within PICs will prove advantageous for applications such as quantum key distribution and cluster-state quantum computing. In addition, by achieving precise control over the placement and coupling of quantum dots within waveguides, these integration techniques are poised to facilitate large-scale implementations in the quantum information field.

### 3.4 Chiral nanostructures

**Figure 6:**
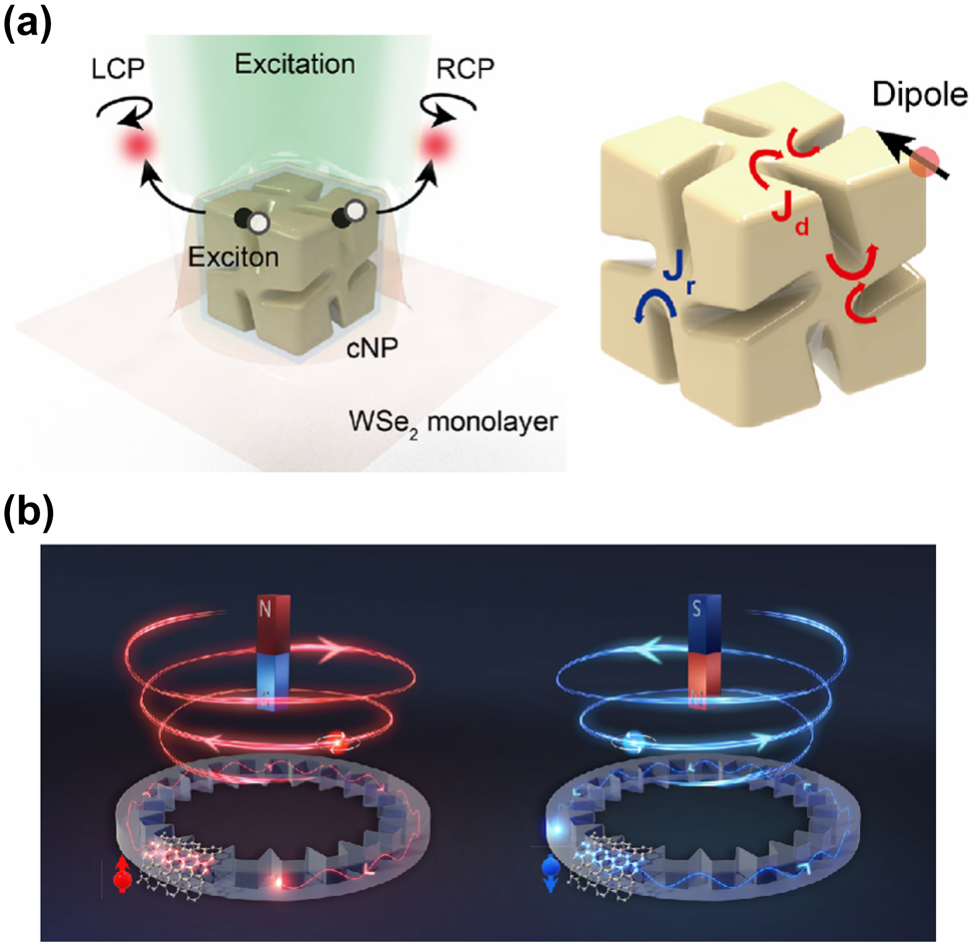
Chiral and twisted QEs. (a) Schematic of chiral quantum emissions from a strain-induced QEs coupled with a chiral nanoparticle. The optical dipole of QE excites the instantaneous induced current, and the retarded induced current. This retardation induces the chiral emission. (b) Schematic of a switchable twisted QEs coupled with a microring resonator that features a pair of quamtum-chiral interfaces at outer and inner sidewalls. By applying an external magnetic field, the intrinsic spin of QE is changed, which leads to switchable twisted QE with a certain OAM topological charge. Reproduced with permission from Ref. [86], American Association for the Advancement of Science, and Ref. [50], American Physical Society.

The integration of chiral nanostructures with QEs to create chiral or twisted single photons has rapidly advanced quantum photonic applications. In this approach, quantum information can be encoded on the single photons with defined spin and orbital angular momentum (SAM and OAM), enabling single photons with spin-dependent, directional propagation, known as spin–orbit locking [50], [51], [52], [124]. In particular, the enhanced light–matter interactions between 2D interfaces and chiral nanostructures have implemented efficient generation of such angular momentum-encoded QEs. For example, SAM-encoded chiral QEs were created by coupling TMD QEs with chiral plasmonic nanoparticles (cNPs) [86]. These cNPs, with helicoidal or asymmetrical structures, introduce localized strain on the 2D material, thereby enhancing emission with defined circular polarization (right or left) (Figure 6a). This configuration enables the controlled emission of chiral single photons, with the chirality dependent on the cNP’s geometry and the QE’s placement.

Meanwhile, the generation of twisted QEs with OAM was demonstrated by coupling QEs with dielectric microring resonators, which support whispery gallery modes (WGMs) that can carry specific OAM states [50]. In this work, the microring’s engineered cross-section supports WGMs with transverse spins, leading to spin-dependent photon propagation around the ring’s edge. With an applied magnetic field, the emitter’s spin-resonant state aligns with either clockwise or counterclockwise modes via spin–orbit locking [52]. Subsequently, the emitted chiral photons interact with an angular grating inscribed at the inner sidewall of the microring. The grating structure converts the circulating photon mode into a twisted photon with a defined OAM (Figure 6b). The generated twisted photon exhibits a doughnut-shaped probability distribution due to a phase singularity at its center, indicative of a nonzero OAM topological charge. This makes it an ideal candidate for encoding quantum states with distinct phase structures. In addition to the high-purity generation of twisted single photons, this platform also features effective on-chip chiral control of quantum light in photonic integrated circuits, facilitating directional single photon routing.

## 4 Conclusions

The deterministic generation and integration of 2D QEs with nanophotonic structures represents an emerging research field with promising prospects but also substantial challenges. Key obstacles include achieving precise emitter placement, control of emission properties, effective coupling with photonic elements such as cavities and waveguides, and a deeper understanding of the photophysics and chemical structure of 2D QEs [16], [78], [80]. Control over the excitonic or the atomic structure and the resultant emission properties remain a core challenge, as spectral inhomogeneities and environmental influence such as thermal, and local strain variations, which can degrade emitter purity and stability [14], [15], [62]. Despite recent advancement, a variety of 2D QEs still limitations in purity, and typically falling short of the high indistinguishability, which are essential for practical quantum photonic applications. Moreover, effective coupling between the QEs and the photonic structures should be achieved. Although plasmonic and dielectric optical systems offer potential for Purcell enhancement, photon guiding, and encoding angular momentum, each has inherent limitations. Plasmonic structures, while enhancing emission through localized surface confinements, also introduce non-radiative decay channels that reduce efficiency [125]. Dielectric structures, on the other hand, exhibit lower losses but generally larger mode volumes, which may reduce coupling strength. The optimization of designs to achieve a balance between these factors remains a significant challenge.

Nevertherless, 2D QEs integrated with nanophotonic structures hold remarkable potential for the development of practical quantum devices, particularly through advances in electrical control [126], [127] and material manipulation [13], [14], [15], [81]. Electrically driven 2D QEs are a promising step toward real-world quantum applications, enabling on-demand, scalable single-photon sources suitable for compact and robust quantum photonic circuits. Moreover, electric and strain-induced tunability affords precise control over emission properties. The recent developments in combining 2D QEs with van der Waals magnets have introduced an exciting new degree of freedom for quantum information processing [128], [129], [130]. By leveraging the intrinsic magnetic properties of these materials, it becomes possible to encode quantum information within spin states, enhancing the functionalities of 2D QEs beyond optical emission alone. This approach promises the development of spin-photon interfaces, which are essential for the implementation of spin-based quantum memory and coherent information transfer across photonic networks [2], [10], [11], [12]. Such hybrid systems showcase the unique ability to manipulate 2D QEs in ways that are not feasible with their bulk counterparts. These include processes such as material manipulation, including stacking and twisting to create moiré superlattices or integrating with other 2D functional materials.
